# Post-Pandemic Resurgence and Seasonal Patterns of Influenza Viruses and Respiratory Syncytial Virus in Arequipa, Peru (2021–2023)

**DOI:** 10.3390/epidemiologia7020057

**Published:** 2026-04-21

**Authors:** Claudia Chipana-Ramos, Ynes Monroy Talavera, Luis Zamudio-Rodriguez, Lucia Villanueva-Sardon, Alexis Germán Murillo Carrasco, Ruy D. Chacón, Yuma Ita-Balta

**Affiliations:** 1 Escuela de Postgrado, Universidad Católica de Santa María, Arequipa 04013, Peru; claudia.chipana@ucsm.edu.pe; 2 Laboratorio de Referencia Regional de la Gerencia Regional de Salud Arequipa, Arequipa 04002, Peru; ynipamela@gmail.com (Y.M.T.); luis.zamudio@ucsm.edu.pe (L.Z.-R.); 3 Facultad de Ciencias Farmaceuticas, Bioquimicas y Biotecnológicas, Universidad Católica de Santa María, Arequipa 04013, Peru; lucia.villanueva@ucsm.edu.pe; 4 Immunology and Cancer Research Group (IMMUCA), OMICS, Lima 15001, Peru; agmurilloc@gmail.com; 5 Pathogen Genetics Research Group (PATHO-GEN), OMICS, Lima 15001, Peru

**Keywords:** influenza A virus, influenza B virus, respiratory syncytial virus, SARS-CoV-2, post-pandemic resurgence, respiratory virus epidemiology, Peru, seasonal distribution, molecular detection, public health surveillance

## Abstract

**Background/Objectives:** The coronavirus disease 2019 (COVID-19) pandemic profoundly disrupted global respiratory virus circulation, with sharp declines during 2020–2021, followed by a resurgence after the relaxation of public health measures. In South America, post-pandemic respiratory virus dynamics remain insufficiently characterized, particularly in ecologically diverse regions. Arequipa, a high-altitude city in southern Peru, has unique environmental conditions, including marked seasonal temperature variability, that may influence viral transmission. **Methods:** We performed a cross-sectional analysis of 21,784 nasopharyngeal swabs collected from symptomatic patients at four major hospitals between June 2021 and September 2023. All samples were tested for SARS-CoV-2 by RT-qPCR. Because routine screening for other respiratory viruses was implemented only in SARS-CoV-2-negative cases during the study period, a subset of SARS-CoV-2-negative samples was subsequently analyzed for influenza A virus (IAV), influenza B virus (IBV), and respiratory syncytial virus (RSV) using VIASURE assays. Viral circulation patterns were evaluated by year, month, and epidemiological week. Meteorological data were obtained from the SENAMHI–La Pampilla station. Logistic regression models were used to assess epidemiological and climatic predictors of viral detection. **Results:** SARS-CoV-2 positivity declined from 20.0% in 2021 to 8.8% in 2023. Conversely, detection of other respiratory viruses among SARS-CoV-2-negative samples increased from 0.8% in 2021 to 29.0% in 2023 (*p* < 0.01). Temporal increases in detection were observed during 2022–2023, particularly for IAV and RSV. In exploratory analyses, calendar year and relative humidity were associated with IAV and RSV detection, while age and temperature variables were associated with IBV. **Conclusions:** Climatic and demographic variables were associated with changes in viral detection for IAV, IBV, and RSV during the post-pandemic transition period in Arequipa. These findings describe patterns of viral detection within SARS-CoV-2-negative symptomatic patients and should be interpreted as surveillance-based observations rather than population-level estimates. Strengthened integrated epidemiological and genomic surveillance will be essential for vaccine planning and outbreak preparedness in the post-pandemic era.

## 1. Introduction

Respiratory viruses are a group of pathogens that affect the respiratory tract and cause illnesses ranging from mild symptoms to severe diseases, such as pneumonia. These viruses are primarily transmitted through aerosols, droplet sprays, direct contact, and fomites [1]. Their modes of transmission disproportionately affect individuals with comorbidities who are exposed to respiratory secretions, direct contact, or contaminated surfaces [2]. Therefore, early detection and epidemiological surveillance are essential to limiting their impact [1,3,4].

In early 2020, the COVID-19 pandemic, caused by SARS-CoV-2, led to an unprecedented global public health response and substantially altered healthcare systems and surveillance strategies worldwide [5]. The widespread implementation of non-pharmaceutical interventions, including mobility restrictions, mask use, and social distancing, significantly reduced overall transmission of respiratory infections [6,7,8]. In parallel, changes in healthcare-seeking behavior and diagnostic priorities affected the detection and reporting of respiratory pathogens [8,9]. Collectively, these and other factors disrupted the baseline epidemiological patterns of respiratory infections during the pandemic period.

The COVID-19 pandemic disrupted the global dynamics of respiratory infections and indirectly affected the circulation of other common viruses [10]. Worldwide, after the relaxation of public health measures in 2022–2023, a resurgence of respiratory diseases caused by viruses, such as influenza and respiratory syncytial virus (RSV), was observed [11]. In Peru, the seasonal peaks of influenza and RSV recorded in 2023 reflect viral reactivation following the easing of restrictions [12]. Competition between SARS-CoV-2 and influenza A and B viruses may partly explain variations in positivity rates and prevalence throughout the year, whereas the transmission of these viruses is also shaped by population immunity, climatic conditions, and public health strategies [13,14,15].

Human influenza is caused by viruses of the *Orthomyxoviridae* family, mainly the genus *Alphainfluenzavirus* (influenza A virus, IAV) and *Betainfluenzavirus* (influenza B virus, IBV), which are associated with pandemics and seasonal epidemics. These viruses exhibit high evolutionary capacity, driven by their segmented genome and elevated mutation rates, which facilitate adaptation to new hosts [16,17,18,19]. Detection is primarily performed using RT-qPCR. For IAV, assays commonly target genes such as hemagglutinin (HA), neuraminidase (NA), and polymerase genes (PB1, PB2), allowing subtype and lineage characterization relevant for surveillance and vaccine formulation [20,21,22]. For IBV, detection focuses on differentiating the B/Yamagata and B/Victoria lineages [23].

Respiratory syncytial virus (RSV), an orthopneumovirus belonging to the family *Pneumoviridae*, is a major cause of acute respiratory infections and hospitalization in infants and older adults. Rapid identification is therefore critical, typically achieved through PCR amplification of the F and G genes, which enables subtype discrimination and supports the epidemiological monitoring necessary for outbreak control [24,25,26,27].

Peru is known for its diverse climatic conditions that may drive distinct viral transmission patterns. Several studies have demonstrated that environmental and climatic factors such as temperature and humidity affect the stability and transmission rates of respiratory viruses [28,29]. Specifically, humidity and temperature have been shown to influence the transmission of SARS-CoV-2 [30], influenza viruses [31], and RSV [32,33]. In this context, Arequipa, a high-altitude city in southern Peru, exhibits relatively mild temperatures throughout the year, marked daily thermal variability, high solar radiation, and a seasonal rainfall pattern characterized by a predominantly dry season and a rainy season with overall low precipitation, without a sharply defined winter period.

This study aimed to assess the positivity rates of influenza A virus, influenza B virus, and respiratory syncytial virus among patients presenting with respiratory symptoms at hospitals in Arequipa, Peru. Using molecular analysis of nasopharyngeal swab samples collected between 2021 and 2023, we examined patterns of viral circulation before and after the relaxation of COVID-19-related public health measures in the context of local climatic variability. This study is explicitly framed as an analysis of a transitional epidemiological period following the COVID-19 pandemic, rather than an effort to characterize long-term or stable seasonal dynamics. Accordingly, the findings should be interpreted within this specific temporal and structural context.

## 2. Materials and Methods

### 2.1. Study Design

A descriptive, non-experimental, cross-sectional study was conducted to analyze the positivity rates of respiratory viruses, including SARS-CoV-2, influenza A (IAV), influenza B (IBV), and respiratory syncytial virus (RSV), in the city of Arequipa, Peru, from June 2021 to September 2023. Although samples were collected over multiple years, the analysis represents repeated cross-sectional observations derived from routine laboratory surveillance rather than a longitudinal cohort design.

The samples consisted of nasopharyngeal swabs obtained from four key institutions in Arequipa: Hospital III Goyeneche (EsSalud), Hospital Regional Honorio Delgado Espinoza (MINSA), Hospital Regional PNP Arequipa (Sanidad PNP), and the Maritza Campos Díaz-Zamacola Health Center (Sentinel Surveillance Center for Influenza). These institutions serve different segments of the population within the Peruvian health system, including the general population receiving care through the Ministry of Health (MINSA), formally employed individuals insured by the Social Health Insurance system (EsSalud), and members of the National Police and their families through the Police Health System (Sanidad PNP).

According to the institutional levels of care, the Maritza Campos Díaz-Zamacola Health Center is classified as level I (mild cases, outpatient management); Hospital III Goyeneche and Hospital Regional PNP Arequipa are classified as level II (moderate to severe cases); and Hospital Regional Honorio Delgado Espinoza is classified as level III (critical cases).

All samples were initially screened for SARS-CoV-2 at the Laboratorio Referencial Regional Arequipa (Peruvian Health Minister, Ministerio de Salud Peruano—MINSA). Subsequently, a representative sample of SARS-CoV-2-negative samples was analyzed to concurrently detect the presence of other common respiratory pathogens, including IAV, IBV, and RSV. This testing strategy reflects the diagnostic workflow implemented during the study period, in which routine screening for non-SARS-CoV-2 respiratory viruses was performed in SARS-CoV-2-negative samples. We report the observed positivity rates and temporal trends of these three viruses. Because detection of influenza A virus, influenza B virus, and RSV was performed only in samples that tested negative for SARS-CoV-2, potential co-infections involving SARS-CoV-2 and these viruses could not be fully assessed and may therefore be underestimated.

### 2.2. Inclusion Criteria and Sample Size

The initial study population consisted of 21,784 nasopharyngeal swab samples collected from patients presenting with respiratory symptoms between June 2021 and September 2023 at the participating hospitals in Arequipa. All samples were routinely tested for SARS-CoV-2 using RT-qPCR as part of the regional diagnostic surveillance program.

During the study period, laboratory testing for other respiratory viruses was implemented as a secondary diagnostic strategy restricted to SARS-CoV-2-negative samples, reflecting the operational workflow used during the COVID-19 pandemic. Consequently, only samples with negative SARS-CoV-2 results were considered eligible for further analysis of other respiratory viruses. Samples were included in the analysis if they met the following criteria: (i) patients presenting with respiratory symptoms (e.g., cough, fever, sore throat, or nasal congestion); (ii) a confirmed negative RT-qPCR result for SARS-CoV-2; (iii) sufficient sample volume for molecular analysis (≥1 mL); and (iv) storage under appropriate refrigeration conditions (≤−20 °C), and analyzed within the within routine laboratory timeframes. All selected SARS-CoV-2-negative samples were processed for IAV, IBV, and RSV detection. Samples were classified as valid, invalid, or inconclusive based on predefined molecular quality control criteria. Samples were considered invalid if the internal control failed to amplify within the expected range, suggesting compromised RNA quality or extraction failure. In addition, samples showing non-specific or non-sigmoidal amplification curves were classified as inconclusive. Only samples with valid amplification profiles were included in regression analyses.

Because testing for IAV, IBV, and RSV was restricted to SARS-CoV-2-negative samples, the findings of this study should be interpreted as positivity rates within the SARS-CoV-2-negative symptomatic population rather than as population-level prevalence estimates for these viruses. This resulted in a sub-universe of 16,992 SARS-CoV-2-negative samples, which constituted the sampling frame for the present study.

From this population, a subset of 2344 samples was selected for the molecular detection of influenza A virus (IAV), influenza B virus (IBV), and respiratory syncytial virus (RSV) using a subset of SARS-CoV-2-negative samples for secondary molecular testing based on operational and logistical capacity within participating laboratories during the study period. The selection of samples for additional testing was not strictly random. Instead, it was influenced by laboratory capacity, reagent availability, and institutional priorities, which varied over time. Consequently, the analytical sample does not represent a proportionally stratified subset of the full SARS-CoV-2-negative population, and temporal imbalances, particularly in 2022, reflect these operational constraints rather than epidemiological patterns

Because the sampling procedure was performed without stratification by year, the analytical sample resulted in a temporally imbalanced sample reflecting operational constraints, which contained a substantially larger number of SARS-CoV-2-negative cases in 2021 than in 2022 and 2023. Consequently, the number of samples analyzed for IAV, IBV, and RSV differed across years, reflecting the structure of the surveillance dataset rather than additional inclusion or exclusion criteria.

The sample size was determined to ensure adequate statistical precision for estimating the positivity rates of respiratory viruses in the SARS-CoV-2-negative population. Assuming a conservative expected proportion of 50%, a 95% confidence level (Z = 1.96), and a finite population of 16,992 eligible samples, a minimum sample size of 2298 samples was required to achieve an estimated margin of error of ±1.83%. The final analytical sample of 2344 samples slightly exceeded this requirement, ensuring sufficient precision for the descriptive and regression analyses performed in this study.

### 2.3. Nucleic Acid Extraction

Nucleic acid extraction was performed on 150 μL of the nasopharyngeal swab sample, which had been previously resuspended in 1 mL of RNase-free water. Nucleic acids were extracted using the Patho Gene-spin™ DNA/RNA Extraction Kit (cat. 17154, Intron Biotechnology, Seongnam-si, Republic of Korea) following the manufacturer’s instructions. The obtained RNA was stored at 2–8 °C until subsequent use. RNA concentration was determined using a Nanodrop One© spectrophotometer (Thermo Fisher Scientific, Waltham, MA, USA), with overall quality evaluation based on 260/230 nm and 260/280 nm ratios.

### 2.4. Real-Time RT-PCR for Respiratory Viruses

Detection of IAV, IBV, and RSV was performed using the VIASURE Viral Influenza A, Influenza B & RSV Positive Control Kit (cat. VS-VP1ABR, CerTest Biotec. Zaragoza, Spain), according to the manufacturer’s recommendations. This multiplex RT-PCR assay has been previously validated and demonstrates high analytical sensitivity and specificity for the detection of these respiratory viruses in clinical samples [34,35].

Detection and amplification were performed using a Rotor-Gene Q real-time thermal cycler (QIAGEN Pleasanton, CA, USA). The target viruses were detected across different fluorescent channels as follows: IAV using the FAM channel (green), IBV using the ROX channel (orange), RSV using the Cy5 channel (red), and the yellow-dye channel was used as the internal control.

Assay validity was ensured by including positive, negative, and extraction controls, as recommended by the manufacturer. A sample was considered positive if it exhibited a sigmoidal amplification curve with a cycle threshold (Ct) value of <40 (Ct < 40). Samples without amplification or with Ct ≥ 40 were classified as negative. In the absence of internal control amplification, samples were classified as invalid, and the analysis was repeated. Samples that did not produce a definitive amplification profile or failed internal control validation were classified as inconclusive (“Unknown”). These samples were reported descriptively but were excluded from regression analyses requiring binary outcome classification.

### 2.5. Epidemiological Data

Epidemiological data for all patients were collected from clinical records and stored in a coded manner to ensure anonymity. Subsequent analyses utilized the following variables from the coded dataset: age (stratified into four groups: <6 years, 6–18 years, 19–65 years, and ≥65 years), sex (male or female), epidemiological week, month, and year of sample collection.

### 2.6. Meteorological Data

To characterize the local climate during the study period, meteorological data were sourced from the public database of the National Service of Meteorology and Hydrology of Peru (Servicio Nacional de Meteorología e Hidrología del Perú, SENAMHI) [36]. Data were obtained from the La Pampilla meteorological station in Arequipa (16°24′49.66″ S, 71°32′4.31″ W, 2326 m above sea level). The parameters considered included the maximum and minimum temperatures (°C), relative humidity (%), and precipitation (mm/day). These variables were calculated as weekly mean values and aligned with the corresponding epidemiological week of sample collection.

### 2.7. Ethical Approval

The study was conducted in accordance with the Declaration of Helsinki and approved by the UCSM Institutional Research Ethics Committee (Dictamen Favorable 353—2025 CIEI-UCSM) on 14 October 2025. The approval was obtained retrospectively for the secondary analysis of anonymized data and samples collected between 2021 and 2023 as part of routine public health surveillance activities. The Ethics Committee reviewed and approved the use of these data for research purposes and granted a waiver of informed consent due to the retrospective nature of the study and the use of de-identified data.

### 2.8. Statistical Analysis

The statistical analysis was structured into descriptive analyses, primary virus-specific regression models, sensitivity analyses, and model diagnostics.

#### 2.8.1. Descriptive Analysis

Data processing began with the calculation of absolute frequencies and positivity rates, which were standardized and expressed per 100 patients evaluated. Differences in positivity rates across the years 2021, 2022, and 2023 were assessed using the chi-squared test (χ^2^).

#### 2.8.2. Logistic Regression Analysis

To evaluate associations between demographic and meteorological variables and virus detection, virus-specific logistic regression models were fitted separately for IAV, IBV, and RSV. The outcome variable in each model was binary (positive/negative).

Multivariable models were applied as an exploratory framework to assess the direction and consistency of associations while accounting for multiple covariates, rather than to infer causality based solely on statistical significance. Given the relatively low number of positive observations for some viruses, particularly the influenza B virus (IBV), the multivariable logistic regression models were interpreted as exploratory analyses intended to identify potential associations rather than to generate precise effect size estimates.

Prior to multivariate modeling, potential collinearity among numerical covariates was assessed using pairwise Pearson correlation analysis, applying a predefined threshold of R > 0.7 to identify strong correlations. To reduce model overparameterization, variables showing statistical significance in the univariate analysis were considered for inclusion in the multivariate models as a screening step [37].

#### 2.8.3. Sensitivity Analysis: Mixed-Effects Logistic Regression

To assess the robustness of the main findings and account for potential temporal clustering, a mixed-effects logistic regression model was fitted as a sensitivity analysis. The outcome variable was defined as the detection of any respiratory virus (IAV, IBV, or RSV). A random intercept for epidemiological week was included to account for shared environmental exposures within each week. A reduced set of covariates (age, calendar year, maximum temperature, and relative humidity) was included as fixed effects to ensure model stability and avoid overparameterization.

Individual-level observations were nested within epidemiological weeks, and a random intercept for epidemiological week was included to account for within-week correlations in environmental conditions.

The epidemiological week was derived from the sampling date using the Sunday-based week definition. The fixed effects included the demographic and environmental variables selected from the univariate screening stage. In the final multivariable model, the following covariates were included: age, calendar year, maximum temperature (°C), and relative humidity (%).

The model was fitted using the Laplace approximation implemented in the glmer() function from the lme4 package in R, with a binomial distribution and logit link. Odds ratios (OR) and 95% confidence intervals (95% CI) were obtained by exponentiating the model coefficients. The variance of the random intercept was examined to evaluate the magnitude of week-level clustering.

#### 2.8.4. Model Diagnostics and Statistical Significance

Model diagnostics were performed to assess the validity of regression assumptions. The Durbin–Watson statistic was used to evaluate potential autocorrelation in residuals. Values close to 2 indicated no significant autocorrelation. The total number of observations included in the regression analyses was 2279 across 45 epidemiological weeks. Logistic regression results are reported as odds ratios (OR) with corresponding 95% confidence intervals (CI). Statistical significance was defined at a confidence level of 95% (*p* < 0.05). All statistical analyses were conducted using IBM SPSS Statistics v.26 and R v4.5.0.

## 3. Results

### 3.1. SARS-CoV-2 Profiling in Arequipa

We initially assessed the overall detection profile of SARS-CoV-2 in the Arequipa population between 2021 and 2023. Table 1 presents the distribution of 21,784 analyzed samples, which represents the complete caseload of suspected viral respiratory illnesses reported to the Arequipa Regional Hospitals.

Of all samples analyzed, 22% were positive for SARS-CoV-2, with the majority of positive cases occurring in 2021 and 2022 (*p* < 0.001). A clear transition was observed in the primary reporting hospital sources during the study period, which reflected changes in sample referral patterns and coverage rather than systematic differences in patient severity or case mix between hospitals. In 2021, Hospital III Goyeneche contributed the highest number of samples for SARS-CoV-2 screening (64%). By 2023, the Policía Nacional del Perú (PNP) Regional Hospital had become the dominant source, contributing approximately half of all suspected cases submitted that year.

### 3.2. Respiratory Viruses Frequency in Negative Samples for SARS-CoV-2 from Arequipa

In this table, the proportion of inconclusive (“Unknown”) results was overall low and showed no consistent pattern across years or hospitals. Notably, no inconclusive results were observed in 2021 for any virus, while a temporary increase was detected in 2022, particularly for IBV and RSV across multiple centers. In contrast, inconclusive results in 2023 were minimal and sporadically distributed. These findings suggest that inconclusive results were not systematically associated with a specific hospital or time period.

Specifically, significant differences were observed between hospitals concerning patient age (both as a quantitative variable and by established age groups), sex, and the relative frequency of the three respiratory viruses detected. Regarding the origin of these samples, Supplementary Figure S1 shows the most representative districts with samples in this study. In addition, we assessed the potential collinearity among all relevant numerical variables included in the study. As shown in Supplementary Figure S2, no strong pairwise correlations were observed, as none of the Pearson correlation coefficients exceeded the predefined threshold (R > 0.7).

Overall, the global positivity rates for the three target viruses were 3.1% for IAV, 1.4% for RSV, and 0.9% for IBV (Table 2).

### 3.3. Respiratory Virus Positivity in Comparison with Weather Factors in Arequipa

Subsequently, we examined the positivity rates of non-SARS-CoV-2 respiratory viruses (IAV, IBV, and RSV) in relation to the local climate profile of Arequipa. Incorporating temporal trend lines into the analysis revealed several potential key associations between the meteorological variables (rainfall, relative humidity, and minimum/maximum temperature). Notably, the temporal patterns of these viruses showed asynchronous peaks, indicating that increases in circulation did not occur simultaneously among the different viruses.

**Figure 1:**
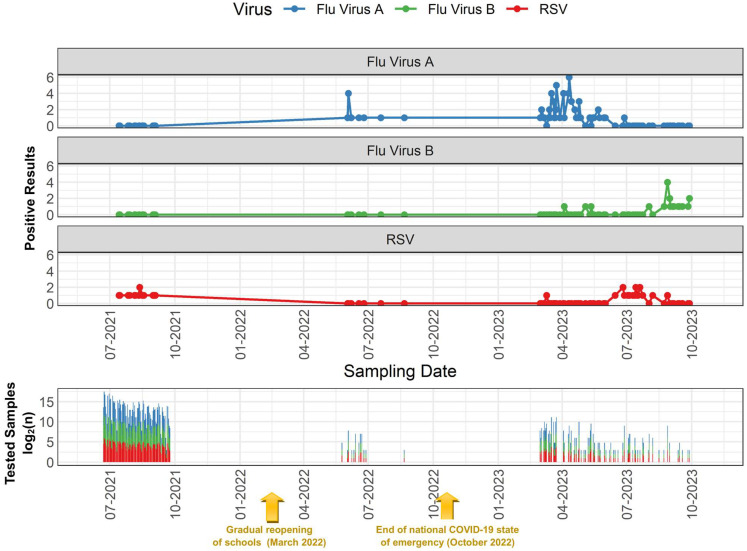
Weekly detection trends of respiratory viruses in Arequipa (2021–2023). This figure illustrates the weekly positivity rates of non-SARS-CoV-2 respiratory viruses detected in respiratory samples collected in Arequipa, Peru, between July 2021 and October 2023. The lines represent the percentage of positive cases by epidemiological week for influenza A virus (blue), influenza B virus (green), and respiratory syncytial virus (red). A stacked bar plot indicates the number of samples processed on each analysis date, providing context for fluctuations in weekly positivity rates. Two gold arrows have been added to provide contextual information on key COVID-19 policy transitions in Peru during the study period, specifically the gradual reopening of schools (March 2022) and the end of the national COVID-19 state of emergency (October 2022).

**Figure 2:**
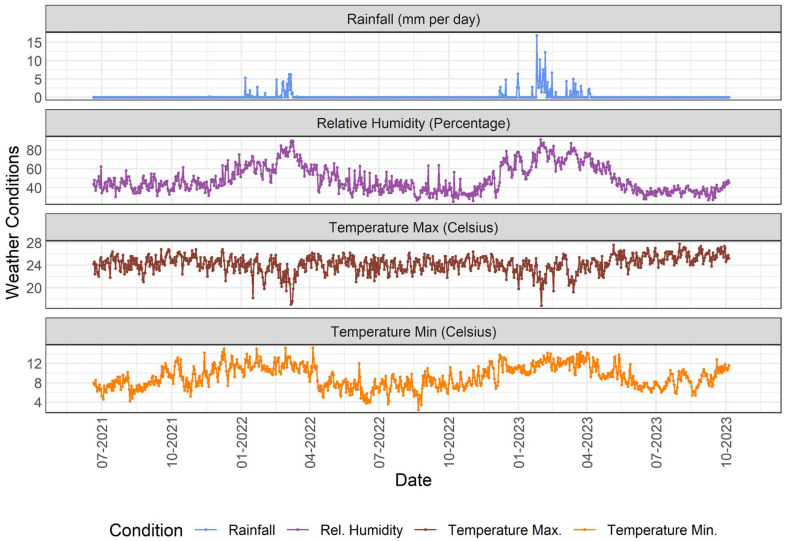
Weekly meteorological conditions in Arequipa (2021–2023). This figure presents the meteorological variables recorded in Arequipa, Peru, during the study period (July 2021 to October 2023). The parameters included rainfall (mm/day), relative humidity (%), maximum temperature (°C), and minimum temperature (°C). The maximum and minimum temperatures corresponded to the highest and lowest daily values recorded by the meteorological station, respectively, and were subsequently averaged by epidemiological week to facilitate comparison with viral detection trends.

Regarding IAV trends, Figure 1 illustrates a significant peak in positive cases observed between July and October 2022, followed by another major peak between April and July 2023. Interestingly, these periods coincided with the dry season in Arequipa, which is characterized by near-zero rainfall and low relative humidity (<45%) (Figure 2).

IBV positivity increased towards the end of the analysis period, specifically between August and October 2023 (Figure 1). This increase coincided with the highest recorded peaks in maximum temperatures in Arequipa (Figure 2). The highest peak in RSV positivity was documented between July and August 2023 (Figure 1). This period coincided with the greatest diurnal temperature variation observed in Arequipa during the study timeframe (Figure 2), as evidenced by the maximum difference between the minimum and maximum recorded temperatures.

### 3.4. Logistic Regression Association Analysis

To individually assess the factors potentially modulating the positivity rates of IAV, IBV, and RSV infections, univariate and multivariate logistic regression analyses were performed. For IAV, all variables evaluated, except sex and season, showed a significant association in the univariate analysis (*p* < 0.05, Table 3). However, following multivariate adjustment, only relative humidity (odds ratio [OR] = 0.931, confidence interval [CI] = 0.867–0.995) and year (OR = 3.810, CI = 1.909–7.947) remained significant (Supplementary Figure S3). Similarly, for IBV, the univariate analysis identified age, relative humidity (Percentage), temperature min (Celsius), temperature max (Celsius), and epidemiological week as potentially associated (*p* < 0.05, Table 3). The subsequent multivariate model refined this to show independent impacts for age (OR = 0.937, CI = 0.906–0.963), temperature Max (OR = 2.344, CI = 1.373–4.230), temperature Min (OR = 1.667, CI = 1.193–2.362), and epidemiological week (OR = 1.111, CI = 1.019–1.227), as shown in Supplementary Figure S4. RSV positivity rates were univariately associated with year, age, relative humidity (percentage), and maximum temperature (*p* < 0.05, Table 3). Of these, the multivariate analysis confirmed year (OR = 1.787, CI = 1.153–2.771), age (OR = 0.939, CI = 0.914–0.961), and relative humidity (OR = 0.936, CI = 0.882–0.984) as significant predictors (Supplementary Figure S5). The minimal dataset used in this analysis is provided as Supplementary Material S1. Because the number of IBV-positive observations in the dataset was limited (n = 20), the corresponding multivariable estimates should be interpreted with caution, as sparse outcome events may produce unstable odds ratio estimates in logistic regression models.

Overall, relative humidity was inversely associated with both IAV and RSV positivity. Year (referring to 2021, 2022, or 2023) consistently demonstrated an influence in the detection of IAV and RSV. Age was inversely associated with IBV and RSV positivity. Furthermore, the estimation of the Durbin–Watson test reported the absence of autocorrelation in the model for IAV (statistic: 1.970; *p* = 0.428), whereas the models for IBV and RSV reported significant *p*-values (*p* < 0.01).

As a sensitivity analysis, a mixed-effects logistic regression model was fitted to account for potential temporal clustering due to shared meteorological exposures across epidemiological weeks. The model included 2279 observations across 45 epidemiological weeks and incorporated a random intercept for epidemiological week. The estimated random-intercept variance was 1.26 (SD = 1.12), indicating measurable week-to-week variability in viral detection.

In the multivariable model, increasing age was associated with lower odds of viral detection (OR = 0.94, 95% CI: 0.91–0.97, *p* < 0.001), whereas calendar year remained positively associated with viral detection (OR = 1.79, 95% CI: 1.11–2.87, *p* = 0.017). Meteorological variables showed weaker associations; maximum temperature was not significantly associated with viral detection (OR = 1.28, 95% CI: 0.82–1.99, *p* = 0.282), whereas relative humidity showed a negative but borderline association (OR = 0.94, 95% CI: 0.87–1.01, *p* = 0.091). This model was based on a pooled outcome (any respiratory virus detection) and was not intended to replace virus-specific analyses. Overall, these findings reflect patterns of viral detection within the sampled population and do not represent population-level incidence or transmission dynamics.

## 4. Discussion

The SARS-CoV-2 pandemic severely impacted Latin America, with Peru being particularly hard-hit. Peru recorded the highest infection-fatality rates, with 660 deaths per 100,000 people and 6604 COVID-19 deaths per million [38,39,40]. Following vaccination campaigns, over 85.76% of Peruvians were fully vaccinated. As public health measures relaxed, other viruses began spreading among individuals with respiratory symptoms. This study examines the shift in respiratory viruses from the COVID-19 pandemic to the post-pandemic period (2021–2023) and explores potential links to climate and demographics. Nevertheless, it is important to consider that the non-random, operationally driven selection of samples for secondary viral testing may have introduced temporal selection bias, particularly underrepresenting certain periods such as 2022.

When COVID-19 emerged in Peru in March 2020, the government implemented one of the world’s strictest lockdowns, including work, travel, and social restrictions, along with mandatory face masks until March 2021 [13]. As vaccination rates increased, restrictions were lifted, and schools and universities reopened by March 2022.

However, social distancing and mask usage persisted until late 2022, when the national emergency concluded [41]. During this period, COVID-19 positivity rates in Arequipa decreased from 30% in 2022 to 8.8% in 2023, with the rise in cases in 2022 primarily attributed to the Omicron variant [42,43]. The pandemic disrupted healthcare services, overwhelming the health system in Arequipa during major epidemic waves (2021 to early 2022). Most COVID-19 tests were conducted at level II hospitals like Hospital III Goyeneche, which had greater capacity, while the Honorio Delgado Regional Hospital (level III) focused on critical cases. After the Omicron wave in 2022, testing became more balanced between level II and III hospitals. By 2023, with fewer COVID-19 cases, testing concentrated on severe cases at Honorio Delgado Regional Hospital and institutional screening at the Peruvian National Police Hospital. This study, though descriptive, illustrates how the Peruvian health system functioned during emergencies and underscores the necessity for well-equipped hospitals to manage high testing demands [44,45].

The study revealed that patient demographics influenced viral infection rates across healthcare centers; however, certain findings were inconclusive, reflecting variability in RNA integrity or sample handling that potentially led to information loss.

The Centro de Salud Maritza Campos Díaz–Zamacola had a higher proportion of young patients, with 45% being children under 18 years old. In contrast, larger hospitals primarily treated adults and older individuals, with children comprising less than 16% of their patients (Table 2). This center exhibited higher rates of IAV (19%), IBV (9.8%), and RSV (5.4%) infections. Notably, most pediatric positive cases were concentrated in this center, accounting for 73.7% (14/19) of IAV, 60% (9/15) of IBV, and 25% (5/20) of RSV cases in individuals < 18 years (Supplementary Material S1). This pattern is consistent with the well-established role of children as key amplifiers of respiratory viruses due to their heightened susceptibility and higher viral loads [17,24,25]. Hospitals with predominantly adult patients had lower infection rates, likely because the virus initially spreads among children [46,47,48]. Similar trends were observed globally, with more children contracting RSV and flu after the pandemic in 2022–2023 [49,50]. One reason for this is that reduced exposure to viruses during social distancing led to lower immunity, creating “immunity gaps” due to decreased exposure during the COVID-19 pandemic [51,52]. These findings highlight the need for health monitoring that considers the patient demographics at each facility.

The timing of respiratory virus outbreaks in Arequipa showed different seasonal patterns for IAV, IBV, and RSV, with each virus having separate epidemic peaks instead of overlapping ones. This pattern fits the idea of viral interference, where infection by one virus temporarily reduces the spread of others due to immune responses [53,54]. This effect has been seen worldwide and may be stronger after the pandemic because of changes in immunity and uneven virus reintroduction. IAV had two main waves: June–August 2022 (peaking at 100% in July) and March–May 2023 (peaking at 100% in April). These occurred under different weather conditions and were caused by different subtypes—H3N2 in 2022 and H1N1pdm09 in 2023—matching reports of influenza changes after COVID-19 restrictions eased [55,56,57]. IBV and RSV each had one main wave: IBV in August–September 2023 (peaking at 75% in September) and RSV in July–August 2023 (peaking at 100% in August), both during dry, cool periods with large temperature swings. Although IBV lineages were not identified here, the B/Yamagata lineage has likely disappeared since 2020, which is important for understanding current IBV trends [58]. These weather conditions support known evidence that cold, dry air with big daily temperature changes helps respiratory viruses survive and spread [29,31]. Still, the different climate conditions for the two IAV waves show that no single environmental factor explains virus resurgence fully. Instead, these patterns result from a mix of seasonal weather and other post-pandemic factors like changing immunity, virus subtype changes, and global immunity gaps [59,60].

A multivariate analysis was done to separate the effects of epidemiological and meteorological factors. The main finding was a clear and significant increase in IAV, IBV, and RSV circulation after the pandemic during 2022–2023. The calendar year was associated with viral detection, especially for IAV and RSV, showing a strong link between the post-pandemic period and increased virus spread. This matches global reports of respiratory virus resurgence after COVID-19 [61,62,63].

IAV was mostly linked only to the year and to lower relative humidity, which supports evidence that dry air helps virus stability and spread through aerosols [29,31]. For IBV, younger age was a moderate risk factor. Unlike IAV, IBV had one late epidemic wave in August–September 2023, coinciding with higher temperatures and greater temperature variation. Previous studies have linked IBV activity to temperature, especially minimum temperature [28,29,31,64], but since similar temperatures occurred at other times without IBV activity, temperature alone likely does not cause IBV outbreaks. The late IBV wave probably results from a mix of climate, chance virus reintroduction, and immunity gaps after the pandemic, so no direct cause can be confirmed from this single event.

RSV showed few links to weather but was more common in younger people and slightly associated with low humidity, similar to IAV. Low humidity may increase RSV spread by helping virus stability, reducing mucous clearance, and aiding aerosol transmission, as seen in other dry or high-altitude regions [32,65,66]. Overall, lower humidity is linked to more virus detection, working together with host factors like age.

These findings show that virus patterns after the pandemic happened alongside changes in population, seasons, and virus circulation over time. Because this study is observational and cross-sectional, the links between weather and viruses should be seen as correlations, not proof that weather causes virus spread, but they fit known seasonal patterns of respiratory virus transmission.

This study has some limitations. First, it used a cross-sectional design due to the public health emergency. It relied on hospital data, which makes it hard to find cause-and-effect relationships or understand how diseases spread in the community. The results focus on patterns over time and associations, not just *p*-values. Differences in healthcare use or testing demand may affect case detection in different hospitals.

Second, the study only looked for IAV, IBV, and RSV in samples that tested negative for SARS-CoV-2. This means it could not find cases where people had both SARS-CoV-2 and these viruses. So, the detection rates are for people with symptoms who tested negative for SARS-CoV-2, not for the whole population. The study might underestimate the total cases of influenza and RSV because it did not check for co-infections. In addition, some regression estimates were unstable due to sparse outcome data and should be interpreted cautiously, particularly for IBV.

It also did not consider factors like mask-wearing or school transmission, which could affect the spread of RSV and IBV. The data did not include details on medical conditions or risk factors like chronic diseases, which could affect how people get respiratory infections. This limits how well the study can adjust for these factors in its analysis. Third, the tests used did not identify specific types of IBV or RSV, and only some influenza subtype information was available. This limits the ability to link outbreaks to specific genetic changes. Fourth, the study did not look at time delays between exposure and symptoms, so the results are based on weekly climate data. Although time-related variables were used, they did not cause issues in the analysis. Despite these limitations, the study provides useful information from the pandemic and post-pandemic periods and can help improve public health strategies in Arequipa and similar areas.

## 5. Conclusions

This study offers a thorough characterization of the post-pandemic resurgence and seasonal reorganization of influenza A virus (IAV), influenza B virus (IBV), and respiratory syncytial virus (RSV) in Arequipa, a high-altitude region where climatic conditions and demographic structure significantly impact viral transmission. The resurgence observed during 2022–2023 unfolded through largely distinct epidemic waves and was linked to demographic factors, such as age, as well as variations in climatic conditions, particularly relative humidity and temperature. These findings are limited to SARS-CoV-2-negative symptomatic patients within the study network and should not be extrapolated to the general population.

Rather than identifying the causal drivers of seasonality, these results outline the temporal patterns of viral detection observed within a hospital-based surveillance network during a post-pandemic transition period. Consequently, the findings should be viewed as exploratory and hypothesis-generating, underscoring the importance of sustained, integrated, year-round surveillance that combines clinical diagnostics, climatic monitoring, and syndromic detection to better anticipate seasonal trends, inform vaccine strategies, and enhance preparedness for future disruptions in respiratory virus circulation. Future studies extending surveillance beyond this transition period are crucial to determine whether the observed patterns stabilize or continue to evolve.

## Figures and Tables

**Table 1:** General profiling of SARS-CoV-2 cases in Arequipa during 2021–2023.

Characteristic	Overall N = 21,784 ^1^	2021 N = 14,301 ^1^	2022 N = 5975 ^1^	2023 N = 1508 ^1^	*p*-Value ^2^
Health Center					<0.001
Centro de Salud Maritza Campos Díaz—Zamacola	169 (0.8%)	143 (1.0%)	1 (<0.1%)	25 (1.7%)	
Hospital III Goyeneche	11,418 (52%)	9105 (64%)	2073 (35%)	240 (16%)	
Hospital Regional Honorio Delgado Espinoza	4301 (20%)	1706 (12%)	2099 (35%)	496 (33%)	
Hospital Regional PNP Arequipa	5896 (27%)	3347 (23%)	1802 (30%)	747 (50%)	
SARS-CoV-2					<0.001
Negative	16,992 (78%)	11,406 (80%)	4210 (70%)	1376 (91%)	
Positive	4792 (22%)	2895 (20%)	1765 (30%)	132 (8.8%)	

^1^ n (%). ^2^ Pearson’s chi-squared test.

**Table 2:** Respiratory virus positivity across different hospital centers in Arequipa, 2021–2023.

Characteristic	Overall N = 2344 ^1^	Centro de Salud Maritza Campos Díaz—Zamacola N = 122 ^1^	Hospital III Goyeneche N = 1258 ^1^	Hospital Regional Honorio Delgado Espinoza N = 406 ^1^	Hospital Regional PNP Arequipa N = 558 ^1^	*p*-Value ^2^
Age	40 (28, 57)	22 (6, 39)	41 (28, 60)	37 (27, 57)	41 (32, 55)	<0.001
Unknown	2	0	2	0	0	
Age (groups)						<0.001
<6 years	128 (5.5%)	28 (23%)	58 (4.6%)	42 (10%)	0 (0%)	
6–18 years	141 (6.0%)	27 (22%)	75 (6.0%)	23 (5.7%)	16 (2.9%)	
19–64 years	1727 (74%)	60 (49%)	893 (71%)	269 (66%)	505 (91%)	
≥65 years	346 (15%)	7 (5.7%)	230 (18%)	72 (18%)	37 (6.6%)	
Unknown	2	0	2	0	0	
Sex						<0.001
Female	1248 (53%)	69 (57%)	705 (56%)	227 (56%)	247 (44%)	
Male	1096 (47%)	53 (43%)	553 (44%)	179 (44%)	311 (56%)	
Year						
2021	1931 (82%)	34 (28%)	1123 (89%)	267 (66%)	507 (91%)	
2022	63 (2.7%)	10 (8.2%)	48 (3.8%)	5 (1.2%)	0 (0%)	
2023	350 (15%)	78 (64%)	87 (6.9%)	134 (33%)	51 (9.1%)	
IAV						<0.001
Negative	2266 (97%)	99 (81%)	1242 (99%)	375 (93%)	550 (99%)	
Positive	73 (3.1%)	23 (19%)	15 (1.2%)	27 (6.7%)	8 (1.4%)	
Unknown	5	0	1	4	0	
IBV						<0.001
Negative	2257 (99%)	101 (90%)	1205 (100%)	393 (99%)	558 (100%)	
Positive	20 (0.9%)	11 (9.8%)	4 (0.3%)	5 (1.3%)	0 (0%)	
Unknown	67	10	49	8	0	
RSV						<0.001
Negative	2248 (99%)	106 (95%)	1196 (99%)	389 (97%)	557 (100%)	
Positive	33 (1.4%)	6 (5.4%)	14 (1.2%)	12 (3.0%)	1 (0.2%)	
Unknown	63	10	48	5	0	

^1^ Median (Q1, Q3); n (%). ^2^ Kruskal–Wallis rank sum test; Pearson’s chi-squared test; NA; Fisher’s exact test. Samples classified as ‘Unknown’ were reported descriptively but excluded from inferential analyses.

**Table 3:** Univariate and multivariate logistic regression analysis of demographic and meteorological factors influencing positivity rates for respiratory viruses in Arequipa, 2021–2023.

		Univariate				Multivariate				
Virus	Variable	OR	Low CI	High CI	*p*-Value	aOR	Low CI	High CI	*p*-Value	*p*-Value ^1^ (Asterisks)
IAV	Epidemiological Week	0.805	0.778	0.831	<0.001	0.877	0.654	1.173	0.376	
	Temperature Min (°C)	1.800	1.629	1.996	<0.001	1.102	0.904	1.353	0.341	
	Relative Humidity (%)	1.096	1.078	1.114	<0.001	0.931	0.867	0.995	0.044	*
	Year	10.437	6.819	18.072	<0.001	3.810	1.909	7.947	<0.001	***
	Rainfall (mm per day)	2.381	1.941	2.920	<0.001	1.354	0.996	1.858	0.055	
	Month (August)	0.002	0.000	0.010	<0.001	0.055	0.000	6.168	0.240	
	Temperature Max (°C)	0.773	0.656	0.914	0.002	0.870	0.646	1.166	0.355	
	Age	0.988	0.976	0.999	0.038	0.995	0.984	1.007	0.440	
	Sex (Male)	0.790	0.488	1.262	0.328					
	Season (Spring)	0.000	0.000	1,228,871.000	0.983					
IBV	Age	0.918	0.889	0.945	<0.001	0.937	0.906	0.963	<0.001	***
	Temperature Max (°C)	3.494	2.180	5.874	<0.001	2.344	1.373	4.230	0.003	**
	Temperature Min (°C)	1.373	1.134	1.640	0.001	1.667	1.193	2.362	0.003	**
	Epidemiological Week	1.184	1.070	1.332	0.002	1.111	1.019	1.227	0.023	*
	Relative Humidity (%)	0.917	0.845	0.981	0.025	0.945	0.852	1.036	0.256	
	Sex (Male)	1.694	0.698	4.338	0.250					
	Season (Spring)	1.813	0.535	8.232	0.376					
	Rainfall (mm per day)	1.152	0.360	1.931	0.701					
	Month (August)	0.766	0.137	14.335	0.803					
	Year	19,681.84	6.45× 10^−11^	NA	0.986					
RSV	Age	0.918	0.895	0.939	<0.001	0.939	0.914	0.961	<0.001	***
	Year	2.632	1.860	3.750	<0.001	1.787	1.153	2.771	0.009	**
	Temperature Max (°C)	1.923	1.407	2.689	<0.001	1.255	0.871	1.833	0.229	
	Relative Humidity (%)	0.895	0.836	0.950	0.001	0.936	0.882	0.984	0.017	*
	Sex (Male)	0.637	0.303	1.281	0.216					
	Season (Spring)	1.831	0.233	37.137	0.601					
	Epidemiological Week	1.013	0.962	1.076	0.642					
	Rainfall (mm per day)	0.890	0.197	1.606	0.793					
	Temperature Min (°C)	0.996	0.812	1.192	0.968					
	Month (August)	1,983,684	1.59 × 10^−13^	1.33 × 10^113^	0.987					

^1^ Statistical reference: (*) *p* < 0.05; (**) *p* < 0.01; (***) *p* < 0.001. Abbreviations: OR, odds ratio; aOR, adjusted odds ratio. Note: Some estimates show extreme values or wide confidence intervals due to sparse data and possible quasi-complete separation.

## Data Availability

The original contributions presented in this study are included in the article and Supplementary Materials. Inquiries can be directed to the corresponding authors.

## Supplementary Materials

The following supporting information can be downloaded at: https://www.mdpi.com/article/10.3390/epidemiologia7020057/s1, Supplementary Material S1: Minimal dataset used in this study, including virus surveillance and meteorological data. Supplementary Figure S1: Distribution of the samples analyzed in this study according to the district of origin. All districts with available information are included. Labels are shown only for districts reporting more than 50 samples. Supplementary Figure S2: Correlation plot (corrplot) displaying Pearson correlation coefficients among the numerical variables analyzed in this study. Color intensity and overlaid coefficients represent statistically significant correlations (*p* < 0.01), whereas blank cells indicate pairwise comparisons that did not reach statistical significance. Supplementary Figure S3: Forest plot showing the contribution of the selected variables in the univariate and multivariate analyses for IAV occurrence. Supplementary Figure S4: Forest plot showing the contribution of the selected variables in the univariate and multivariate analyses for IBV occurrence. Supplementary Figure S5: Forest plot showing the contribution of the selected variables in the univariate and multivariate analyses for RSV occurrence.
