# Supplementary figures and images for: Post-Pandemic Resurgence and Seasonal Patterns of Influenza Viruses and Respiratory Syncytial Virus in Arequipa, Peru (2021–2023)
Source: Epidemiologia (Basel). 2026 Apr 21;7(2):57. doi: 10.3390/epidemiologia7020057 (PMC13115137; doi:10.3390/epidemiologia7020057)

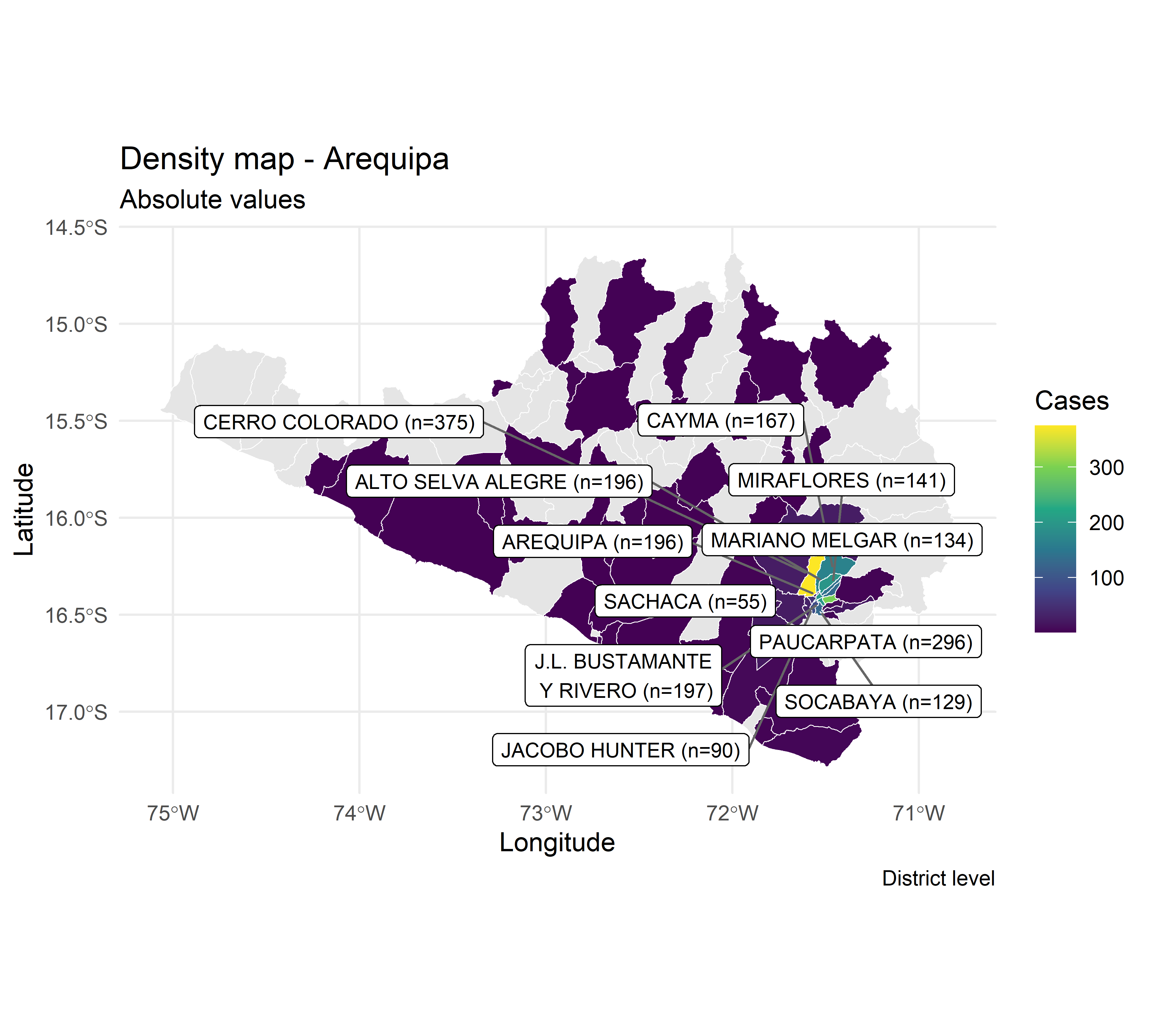

Supplement: Supplementary file 1 [file epidemiologia-07-00057-s001.zip › Supplementary Figure 1.png]

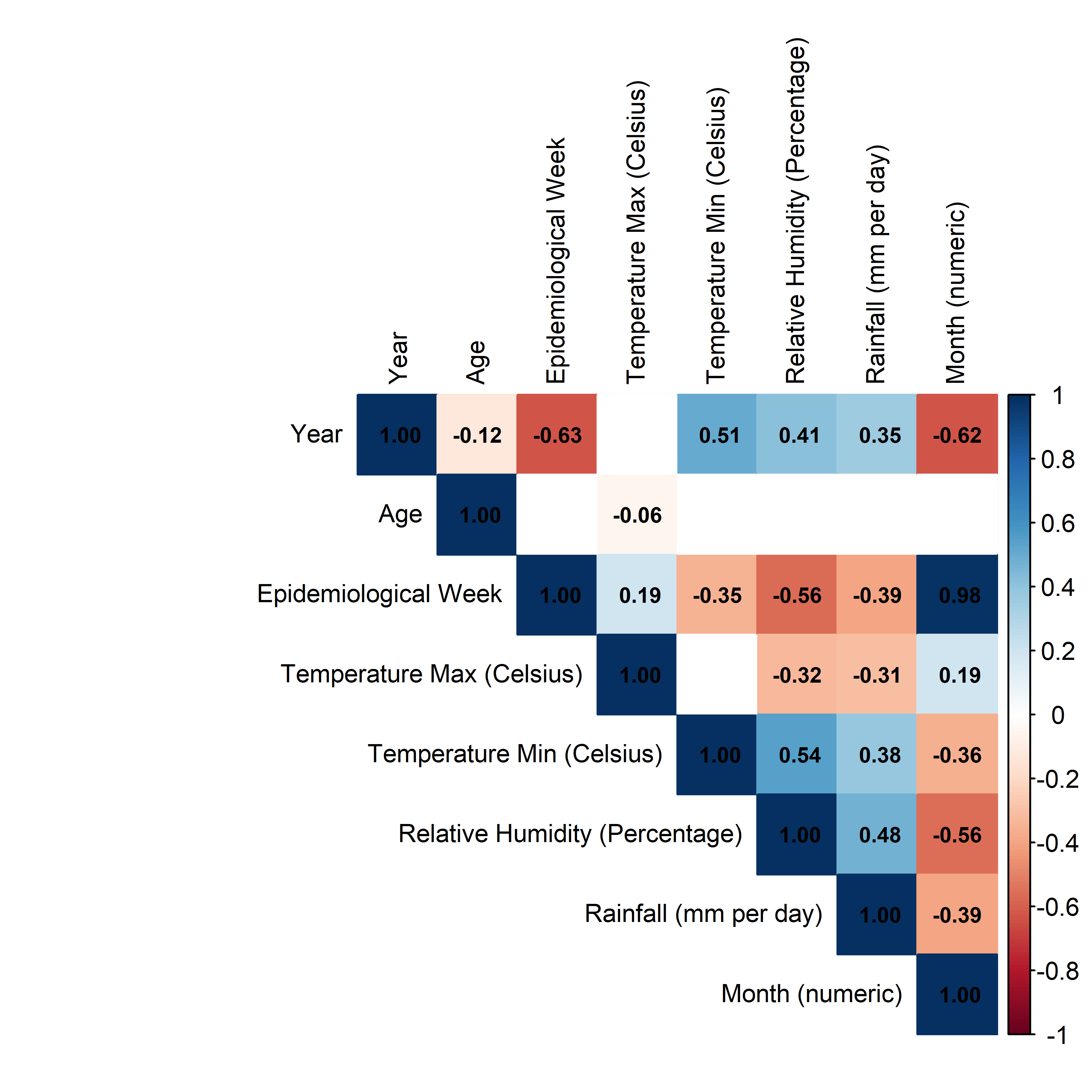

Supplement: Supplementary file 1 [file epidemiologia-07-00057-s001.zip › Supplementary Figure 2.png]

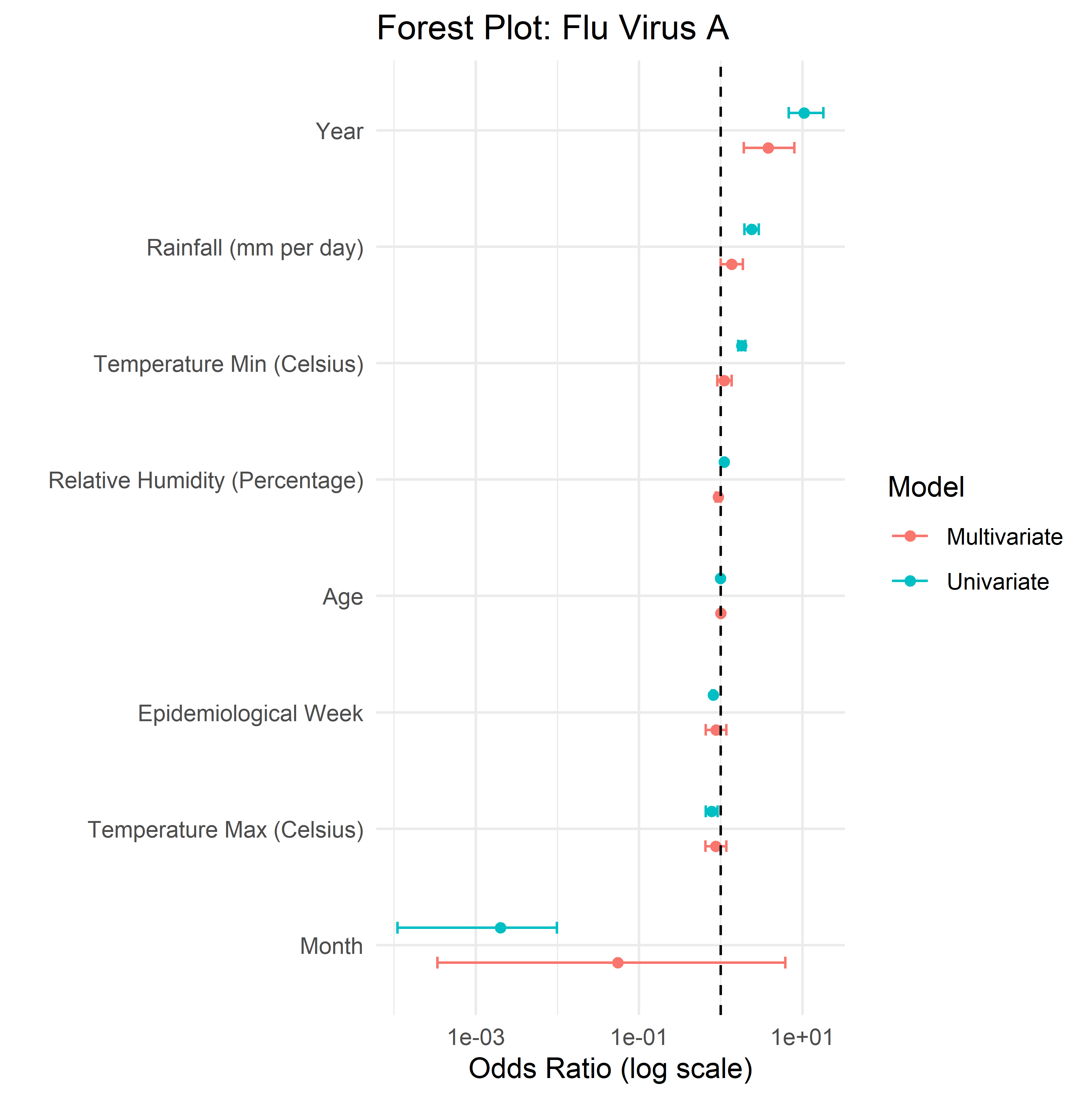

Supplement: Supplementary file 1 [file epidemiologia-07-00057-s001.zip › Supplementary Figure 3.png]

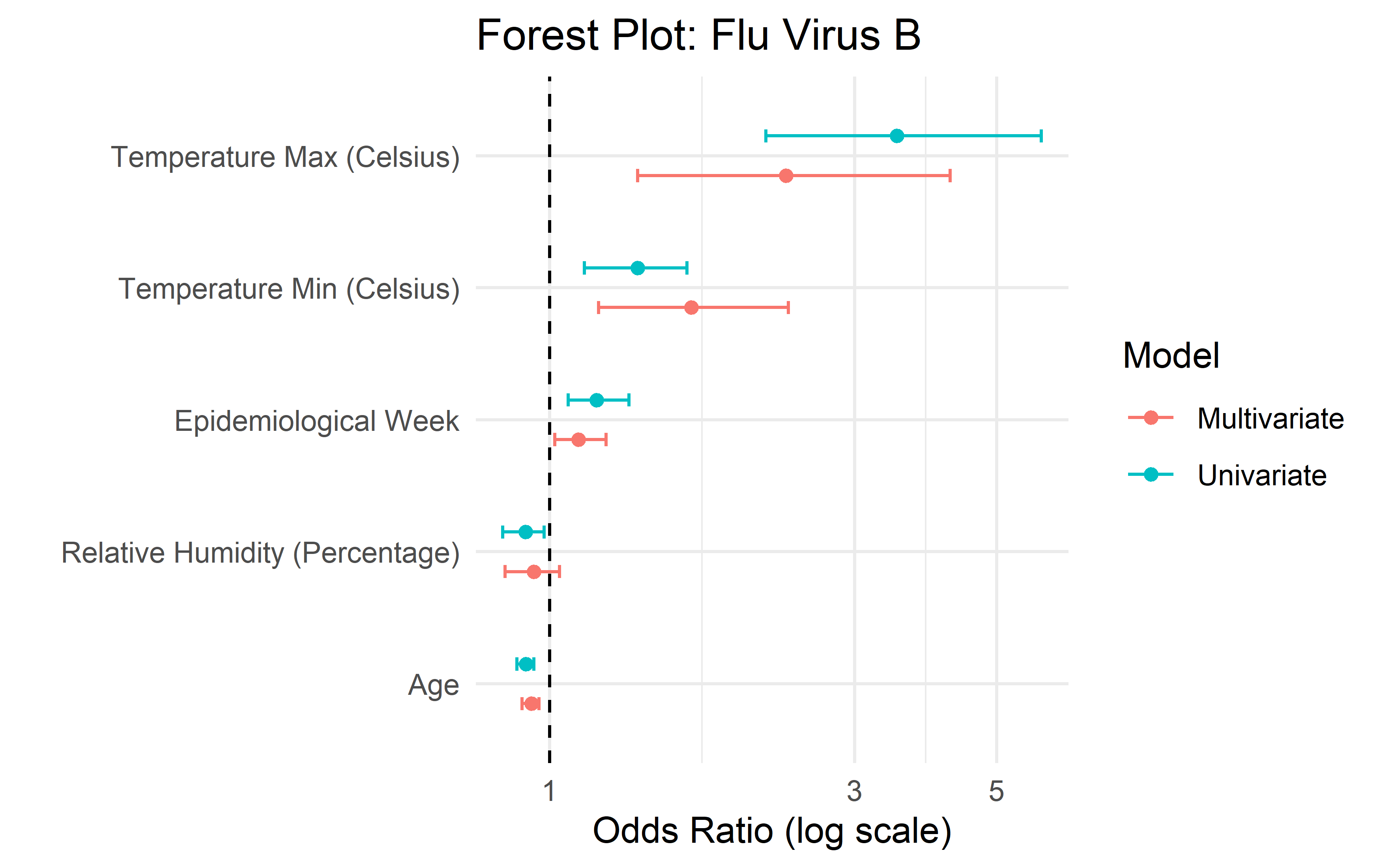

Supplement: Supplementary file 1 [file epidemiologia-07-00057-s001.zip › Supplementary Figure 4.png]

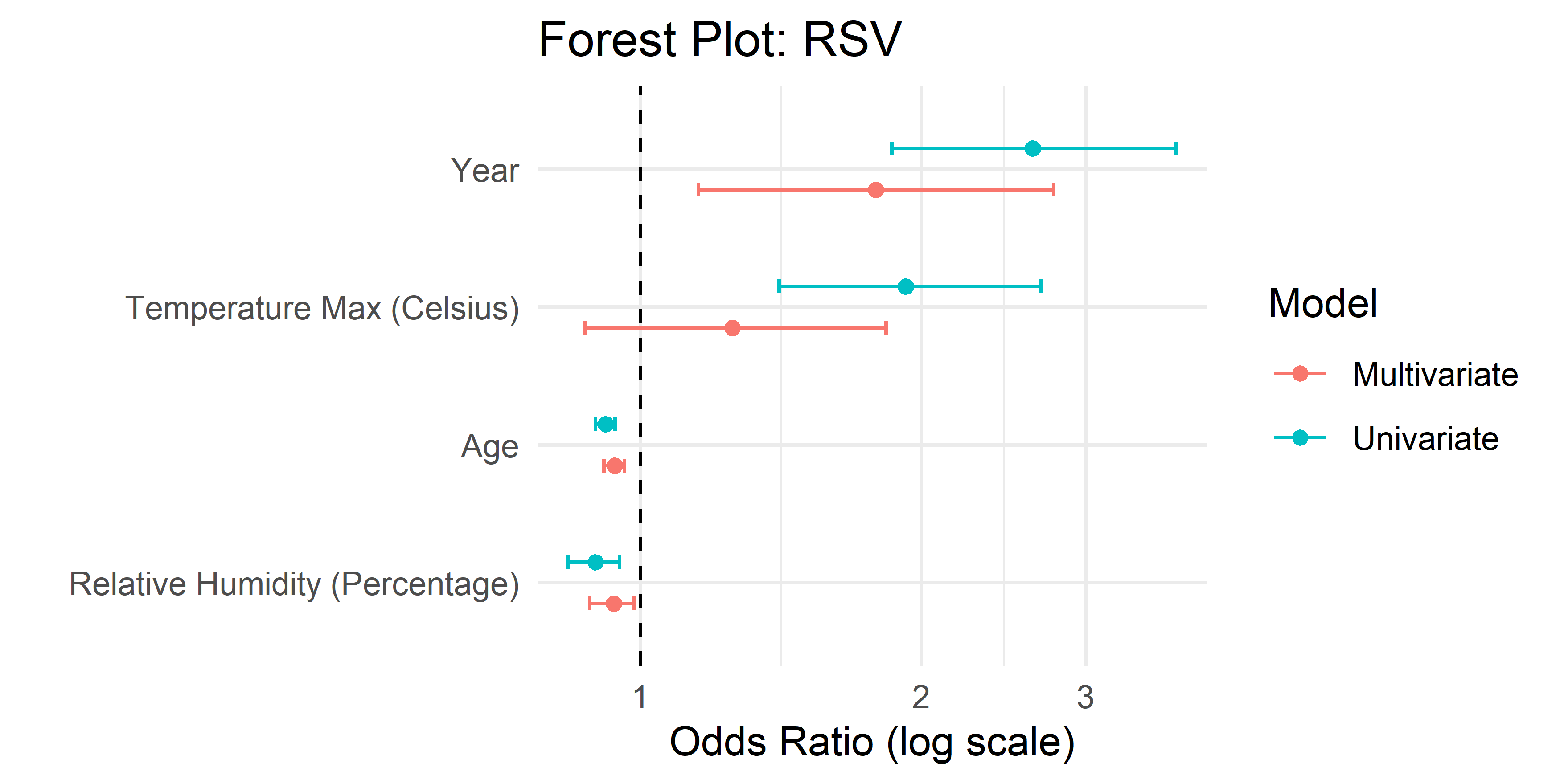

Supplement: Supplementary file 1 [file epidemiologia-07-00057-s001.zip › Supplementary Figure 5.png]
